# Correction: Maternal Cypermethrin Exposure during the Perinatal Period Impairs Testicular Development in C57BL Male Offspring

**DOI:** 10.1371/journal.pone.0105668

**Published:** 2014-08-11

**Authors:** The PLOS ONE Staff

There are errors in the Funding section. The correct funding information is as follows:

This work was supported by the China 973 Project (Grant No. 2009CB941701) and National Natural Science Foundation of China (Grant No. 31071316) to XL. The funders had no role in study design, data collection and analysis, decision to publish, or preparation of the manuscript.
